# A 3D “sandwich” co-culture system with vascular niche supports mouse embryo development from E3.5 to E7.5 in vitro

**DOI:** 10.1186/s13287-023-03583-2

**Published:** 2023-12-10

**Authors:** Junjun Xu, Linye Zhang, Zihui Ye, Binwen Chang, Zheng Tu, Xuguang Du, Xi Wen, Yili Teng

**Affiliations:** 1 School of Basic Medical Sciences, Wenzhou Medical University, Wenzhou, 325015 China; 2 The First School of Medicine, School of Information and Engineering, The First Affiliated Hospital, Wenzhou Medical University, Wenzhou, 325015 Zhejiang China; 3 School of Public Health and Management, Wenzhou Medical University, Wenzhou, 325015 Zhejiang China; 4 Renji College, Wenzhou Medical University, Wenzhou, 325015 Zhejiang China; 5 State Key Laboratory of Animal Biotech Breeding, College of Biological Sciences, China Agricultural University, Beijing, 100193 China; 6 Department of Gynecology and Obstetrics, Xuanwu Hospital, Capital Medical University, Xicheng District, Beijing, 100053 China; 7 Reproductive Medicine Center, The First Affiliated Hospital, Wenzhou Medical University, Wenzhou, 325015 Zhejiang China

**Keywords:** 3D “sandwich” co-culture system, Vascular niche, E3.5–E7.5 development

## Abstract

**Background:**

Various methods for ex utero culture systems have been explored. However, limitations remain regarding the in vitro culture platforms used before implanting mouse embryos and the normal development of mouse blastocysts in vitro. Furthermore, vascular niche support during mouse embryo development from embryonic day (E) 3.5 to E7.5 is unknown in vitro.

**Methods:**

This study established a three-dimensional (3D) “sandwich” vascular niche culture system with in vitro culture medium (IVCM) using human placenta perivascular stem cells (hPPSCs) and human umbilical vein endothelial cells (hUVECs) as supportive cells (which were seeded into the bottom layer of Matrigel) to test mouse embryos from E3.5 to E7.5 in vitro. The development rates and greatest diameters of mouse embryos from E3.5 to E7.5 were quantitatively determined using SPSS software statistics. Pluripotent markers and embryo transplantation were used to monitor mouse embryo quality and function in vivo.

**Results:**

Embryos in the IVCM + Cells (hPPSCs + hUVECs) group showed higher development rates and greater diameters at each stage than those in the IVCM group. Embryos in the IVCM + Cells group cultured to E5.5 morphologically resembled natural egg cylinders and expressed specific embryonic cell markers, including Oct4 and Nanog. These features were similar to those of embryos developed in vivo. After transplantation, the embryos were re-implanted in the internal uterus and continued to develop to a particular stage.

**Conclusions:**

The 3D in vitro culture system enabled embryo development from E3.5 to E7.5, and the vascularization microenvironment constructed by Matrigel, hPPSCs, and hUVECs significantly promoted the development of implanted embryos. This system allowed us to further study the physical and molecular mechanisms of embryo implantation in vitro.

**Supplementary Information:**

The online version contains supplementary material available at 10.1186/s13287-023-03583-2.

## Introduction

Embryogenesis in the maternal uterus is an extremely sophisticated and intricate process involving multilevel regulation. The oosperm undergoes cleavage several times after fertilization and generates small blastomeres whose fates are determined by two lineage choices, subsequently founding embryonic and extra-embryonic lineages [1]. The morula progressively develops into a hollow spheroid, termed the blastocyst, during cleavage. A pivotal step in mammalian embryonic development is blastocyst implantation into the maternal uterine wall, establishing the foundation for subsequent development [2, 3]. Mouse embryo implantation occurs in E4.5, when the blastocyst is composed of primitive endoderm (PE), epiblast cells (EPI), and trophectoderm (TE) [4]. The TE and the inner cell mass (ICM) are distinguished at the first decision determining cell fate; the TE forms the placenta, while the ICM retains pluripotency, which generates PE and EPI in the second cell fate decision [5, 6]. Epiblast cells represent the cell lineage that forms all body tissues in the future, whereas PE is another extra-embryonic lineage that evolves into the yolk sac [7]. Implantation implies that the embryo gradually exits pluripotency and initiates cell specification [8]. The formation of the embryonic cavity is triggered by EPI polarization [9]. Embryonic cells undergo rearrangements after implantation, resulting in the development of the disorganized EPI into the polarized cup-shaped epithelium located at the distal part of the embryo [3]. Thereafter, the blastocyst transforms into an epiblast rosette with cavities at E5.0 [4]. The blastocyst elongates from E4.5 to E5.5, undergoes lumenogenesis to transform into the egg cylinder, and the PE develops into the visceral endoderm (VE). The VE comprises two parts: the distal visceral endoderm (DVE) and the anterior visceral endoderm (AVE), which are assembled by the partial DVE that migrates to the prospective anterior side at E6.5 [10]. The first critical morphogenetic event in post-implantation development occurs during DVE migration, wherein cavities of the extra-embryonic ectoderm (ExE) and EPI unite into one structure called the pro-amniotic cavity [4, 11]. The primitive streak appears on the posterior side, which is adjacent to the ExE [12]. The embryo breaks the symmetry, establishes the anterior–posterior axis, and originates gastrulation [13]. Gastrulation of the mouse embryo involves the rearrangement of cell lineages, which eventually results in the formation of differentiated tissues and organs [14, 15].

The mammalian embryo builds up the body plan shortly after implantation [16]. However, studying and manipulating the embryo is difficult since the natural embryo cannot be observed or directly obtained in utero [17]. Most research since the 1950s has been devoted to exploring systems to culture mouse embryos in vitro; the transfer of in vitro cultured blastocysts into the uterus of pseudopregnant mice results in normal development and successful delivery of the offspring [18, 19]. A variety of platforms and media for *ex utero* development are established, such as a rat serum culture medium composed of 80% male rat serum with 20% Hanks’ balanced salt solution and a serum-free medium comprising N-2 and B-27 supplements [20, 21]. In addition, a static system fosters embryos on chick plasma clots [22], a circulating medium fosters and observes embryos in a circulator [23], and a roller culture system fosters embryos on a drum [16]. It is a great achievement that mouse embryos can be stably cultured from pre-gastrulation (E5.5) to late organogenesis (E11) in vitro. However, although researchers attempt to culture pre- and post-implantation mouse embryos in vitro, the approaches of culture from the blastocyst (E3.5) to the gastrula (E7.5) and subsequent embryonic development is still not established [16].

Researchers have attempted to test approaches to culture embryonic stem cells (ESCs) in vitro since they are relatively easy to obtain and study. Aggregates of ESCs are termed embryoid bodies (EBs) [24], and they can self-organize in suspension culture [25]. However, EBs cannot imitate embryonic development or recapitulate gastrulation. A new embryonic model was established in 2014 using murine embryonic stem cells (mESCs). The formation of embryonic models (“gastruloids”) was developed by culturing mESCs using adherent culture with N2B27 for 2 days, followed by successive treatment with activin A (Act) and CHIR99021 (CHI) (an agonist of Wnt/β-catenin signaling) [25, 26]. Three-dimensional gastruloids are aggregates of mESCs, and several key events in embryogenesis can be recapitulated in vitro*,* such as self-organization into polarized structures, symmetry breaking, body axis formation, and elongation [26, 27]. Thus, Sozen et al. [13] showed that co-culturing ESCs, trophoblast stem (TS) cells, and extra-embryonic-endoderm (XEN) cells in vitro (and allowing their self-organization without interfering with the arrangement of cells) results in these cell lineages spontaneously forming ETX (ESC + TS + XEN) embryos. However, gastrulation in ETX embryos is not integrated. Amadei et al. [28] developed induced ETX embryos (iETX embryos) composed of TSCs and ESCs that transiently express the transcription factor, Gata4; iETX embryos possess the greater developmental potential for breaking symmetry and directing the migration of AVE. The absence of anterior neural tissues in gastruloids is an urgent problem compared to natural embryos in vivo; thus, hydrogel microwell arrays and high-throughput cell aggregation were used to promote the development of mESCs into EPI-like aggregates, which are propitious to the formation of brain-like regions and anterior neural tissues [29, 30]. In addition, gastruloids can develop into neural tubes [31], somites [31, 32], and beating heart-like structures [33]. Recently, Amadei et al. gathered stem cells derived from mESCs, TSCs, and inducible XEN (iXEN) cells to generate ETiX embryoids that reconstitute normal embryonic development in vivo up to E8.5. Furthermore, they can potentially form organs or tissues, including definite forebrain and midbrain regions, beating heart-like structures, neural tubes and somites, and the gut [34]. Lau et al. created EiTiX-embryoids, an *ex utero* embryonic model based on ESCs cells. The model establishes the anterior–posterior axis and undergoes pre-gastrulation to neurulation stages, with the appearance of tissues such as headfolds and the brain. Single-cell RNA sequencing reveals partial concordance between EiTiX and mouse embryos in vivo [35]. Furthermore, a 3D differentiation system is established to induce the formation of extended pluripotent stem (EPS) cells to form blastoids through self-organization and lineage separation [36]. Mouse embryonic EPS cells and TSCs containing EPI, PE, and TE incorporate and self-organize into EPS-blastoids, similar to late blastocysts in natural embryos. EPS-blastoids can mimic the pre- to post-implantation transition in normal embryonic development in vitro and undergo successful implantation into the uterus of pseudopregnant mice [37].

Whole embryo culture platforms were established to more accurately determine the developmental processes of mammalian embryos [16, 20]. Furthermore, embryo-like structures with pluripotent stem cells that can imitate natural development were constructed, such as EBs [24] and gastruloids [26]. However, such systems and models are simple simplifications of developmental conditions and processes in vivo. Despite many attempts, it is still impossible to replicate the uterine environment and deliver healthy baby mice in vitro. Currently, gastruloids are widely regarded as the most advanced post-implantation models [31], but they cannot recapitulate the interactions of signaling pathways between cell lineages and capture all the intricate signals and key morphological events throughout embryogenesis. Previous trials show that gastruloids cannot represent the complete embryo.

Mouse embryos undergo consecutive and complex development in vivo following stimulation by factors secreted by the endometrium. Synchronous preparation of the endometrium is a requisite for implantation during embryonic development. Evans et al. [38] developed an in vitro model based on TE to simulate the pre-implantation human embryo and confirmed that endometrial extracellular vesicles (EVs) improve the adhesion and invasion capacity of TE, which plays an essential role in implantation. Gurung et al. [39] also showed that human endometrial epithelium-derived exosomes could affect the TE of blastocysts to enhance the possibility of implantation. Vascularization (the emergence of new blood vessels) determines whether embryos can continue subsequent development, such as placenta formation [40]. Following implantation, mouse embryo trophoblast giant cells migrate and collectively penetrate the uterine stroma to express vascular receptors, ligands, and adhesion molecules to interact with maternal blood vessels, which triggers vascularization [41, 42]. Endometrial mesenchymal stromal cells (endMSCs) are pluripotent and can promote regeneration. Mouse embryos are cultured in vitro with different concentrations of extracellular vesicles derived from endMSCs (EV-endMSCs) to test the conditions for normal development in vitro*.* However, no evidence exists that EV-endMSCs manifested by increased blastomeres are conducive to embryonic development [43]. Therefore, we constructed an effective in vitro system (a three-dimensional (3D) “sandwich” co-culture system) wherein human placenta perivascular stem cells (hPPSCs) and human umbilical vein endothelial cells (hUVECs) were seeded to the bottom layer to promote the normal developmental rates of embryos from the early blastocyst stage (E3.5) to the late gastrula stage (E7.5). It was ascertained that embryos could complete the morphological transition from pre- to post-implantation, given the effects of hPPSCs- and hUVEC-secreted factors in vitro and exhibit higher rates of normal development and greater diameters in each period. Embryos grown in vitro to the pre-gastrula stage (E5.5) transferred to the uterus of pseudopregnant mice maintained the potential to implant and continue developing until a certain stage.

## Materials and methods

### Animals and human cells

Six-seven-week-old female and male Kunming (KM) mice and 9-11-week-old male KM mice were obtained from Zhejiang Vital River Laboratory Animal Technology Co., Ltd. Females were randomly divided into 34 groups (4 mice/group, 34 groups of 136 mice), and randomization was achieved using an online random number generator (https://www.graphpad.com/quickcalcs/randomize1/). Mice were housed under a 12 h light–dark cycle at 19–22 °C with free access to food and water. A weight loss > 25% was defined as a humane endpoint. No humane endpoint was met, and adverse events in mice were not observed during the study. Our manuscript is reported following the ARRIVE guidelines (https://arriveguidelines.org/arrive-guidelines). Human placenta perivascular stem cells were isolated from the placenta, and hUVECs were from the human umbilical cord.

### In vitro culture medium

The embryo in vitro culture medium (IVCM) was modified according to previously described methods [3, 11, 16, 44–46]. It was composed of 23% Dulbecco's modified Eagle’s medium (DMEM) with high glucose (v/v) (Adamas Life, C8013) supplemented with 70% heat-inactivated (HI) horse serum (v/v) (Gibco, 26050088), 11 mM HEPES (Solarbio, H1095), 2 mM GlutaMax (Gibco, 35050-061), 1% penicillin–streptomycin (v/v) (Gibco, 15140-122), 100 µM minimal essential medium (MEM) non-essential amino acids (NEAA) (Gibco, 11140-050), 55 µM 2-mercaptoethanol (Gibco, 21985-023), 1% ITS-X (v/v) (Gibco, 51500-056), 8 nM β-estradiol (Sigma, HY-B0141), 200 ng/ml progesterone (Sigma, HY-N0437), 25 µM N-acetyl-L-cysteine (Sigma, HY-B0215), 0.2% N-2 supplement (v/v) (Gibco, 17502048), and 0.9% B-27 supplement (v/v) (Gibco, 17504044).

### In vitro embryo recovery and culture

Female KM mice were superovulated by intraperitoneal injection of 10 U pregnant mare’s serum gonadotropin (PMSG) (Ningbo Second Hormone Factory, 110254564) for 48 h, followed by the injection of 10 U human chorionic gonadotropin (hCG) (Ningbo Second Hormone Factory, 110251283). Animals were then mated with KM males at a 1:1 ratio. The following day, females were inspected for vaginal plugs, and noon was defined as E0.5 d post-coitum (dpc). Female mice with plugs were were donor mice (E0.5).

Donor mice were anesthetized with pentobarbital sodium (Shanghai EKEAR Biotech, 57-33-0) (0.1 ml/10 g, i.p.) and killed by cervical dislocation at E3.5 to collect E3.5 embryos. The skin and peritoneum were cut, and the uterus was dissected from the mesometrium and transferred to warm normal saline (Zhejiang Guojing Pharmaceutical Co., Ltd., C21031704). The uterus was injected from one uterine horn with warm M2 medium using a 27-Gauge syringe under a dissecting microscope (Tansoole, 02026616-TS069-004). The recovered embryos were transferred into drops of M2 medium after uterine flushing under an inverted microscope (EmbryoMax M2 Medium with Phenol Red, Emd Millipore Corp., MR-015-D) using a mouth pipette to remove impurities. Embryos were washed three times in drops of M2 medium and then three times in drops of IVCM. Subsequently, the embryos were randomly assigned and transferred to the middle layer of culture wells.

A 3D culture system of embryos was established using the culture wells of a 96-well plate. E3.5 embryos were cultured in IVCM (the IVCM system) or IVCM-added hPPSCs and hUVECs (the IVCM + Cells system). The bottom layer comprised 35 µl Matrigel (Corning, 354277) mixed with 25 µl IVCM. The middle layer was composed of 10 µl Matrigel mixed with 25 µl IVCM, and the upper layer was composed of 100 µl IVCM. Embryos were seeded into the middle layer, and feeder cells (hPPSCs and hUVECs) were seeded into the bottom layer. The density of hPPSCs and hUVECs was 3.3 × 10^5^ cells/ml. The components of the bottom layer were added to a culture well and incubated at 37 °C for 20 min until the Matrigel solidified. Embryos were seeded and incubated at 37 °C for 20 min after the components of the middle layer were added. Then, 100 µl of IVCM was added to the culture well. Embryos were placed in a constant temperature incubator under 5% CO_2_ at 37.5 °C. Half of the medium in the upper layer was replaced with fresh medium every 24 h.

### Immunofluorescence of embryos and hPPSCs

Embryos in the culture well were transferred into a drop of 60 µl 1% bovine serum albumin (BSA) (Beyotime, ST025-29g). Impurities were removed from embryos by washing them once in 1% BSA drops using a mouth pipette under an inverted microscope. Embryos were fixed in 4% paraformaldehyde (Jiangsu Yonghua Fine Chemical Co., Ltd., 30525-89-4) for 20 min at 25 °C, washed twice in 1% BSA, and permeabilized in 0.5% Triton X-100 (Sigma-Aldrich, 066K0089) for 1 h at 25 °C. Embryos were washed with 1% BSA, followed by incubation with a primary antibodies diluted in 1% BSA for 1 h at 25 °C and washed twice with 1% BSA. Embryos were then incubated with secondary antibody diluted in 1% BSA for 30 min at 25 °C, washed twice with 1% BSA, and transferred to 4′,6-diamidino-2-phenylindole (DAPI) on a microscope slide. The slide was then overlaid with a coverslip and sealed with nail polish. Staining results were observed using a fluorescent inverted microscope. The primary antibodies for embryo immunofluorescence were rabbit anti-OCT4 polyclonal antibody (1:200, Thermo Fisher Scientific, PA5-27438), rabbit anti-Nanog antibody (1:100, Thermo Fisher Scientific, PA5-85110), Rabbit anti-Cdx2 antibody (1:1000, Abclonal, A19030), Goat anti-Gata4 polyclonal antibody(1:100, R&D system, BAF2606), Goat anti-Sox17 polyclonal antibody(1:100, R&D system, AF1924), Goat anti-T/Brachyury polyclonal antibody(1:100, R&D system, AF2085).The secondary antibody was FITC-goat anti-rabbit IgG (1:500, Thermo Fisher Scientific, F-2765), Donkey anti-goat IgG H&L (Alexa Fluor® 488) (1:500, Abcam, ab150129).

### Uterus transfer of E5.5 embryos cultured in vitro

Superovulated female KM mice were mated with vasectomized KM males at 1:1 to prepare pseudopregnant surrogates. The following morning, females with plugs were deemed E0.5 pseudopregnant mice.

The surrogate was anesthetized at E3.5, and the uterus was exposed. E3.5 embryos from the culture well were transferred to the M2 medium. Embryos were washed twice with M2 medium using a mouth pipette and then transferred into a uterine horn punctured with a 27-Gauge needle, and the muscle incision and skin were sutured. Approximately 15 embryos were transferred to each uterine horn. The uterus and ovary were dissected 4 days post-transfer, and embryo implantation was observed.

### Human umbilical vein endothelial cells and hPPSCs co-cultured on 3D Matrigel

Reduced growth factor basement membrane extract (RGF BME) (R&D Systems, 3433-005-01) was thawed on the ice at 4 °C. A 48-well plate was pre-warmed at 37.5 °C, while 1.5-ml microcentrifuge tubes and P200 pipette tips were pre-chilled at 4 °C. Basement membrane extract was transferred to a 48-well plate and incubated at 37 °C for 20 min. GFP-hUVECs and mCherry-hPPSCs reached 70–80%, and confluence was added on BME, 37.5 °C, 5% CO_2_.

### Statistical analysis

Quantitative data were presented as the mean ± standard deviation. Statistical tests were performed using IBM SPSS Statistics 25 software. First, skewness value/std. error of skewness and kurtosis value/std. error of kurtosis were used to check the normality of the data distribution. The data conformed to normality if both skewness value/ std. error of skewness and kurtosis value/std. error of kurtosis were < 1.96(Absolute value). Levene's test was used for homogeneity of variances, and *P* > 0.1 was determined as homogeneous. Two samples were compared using an independent sample. Student’s *t*-test, where *α* = 0.05, *P* < 0.05, indicates that the data were statistically significant. When the variance was unequal, the *t*′ test was applied (SPSS degrees of freedom and t values corrected). Multiple groups of independent samples were compared using one-way analysis of variance (one-way ANOVA): *α* = 0.05 when the data met the conditions of normality, independence, and homogeneity of variance (*P* > 0.1). The rank-sum test was used if the homogeneity of variance was not met (*P* < 0.05 was regarded as statistically significant). Graphs were generated using GraphPad Prism 8.0.1. Statistical analyses were performed of normal embryo developmental rates and embryo length. *n* was used to refer to the total number of replicates of the experimental or control groups; the *n* value in each experiment was determined considering data accuracy, available resources, and animal ethical constraints of female mice use. Allocation of experimental units (*n*) is found in Supporting information 1 and Supporting information 2; *n* refers to the numbers of embryos studied during statistical analyses of the implantation rates and the immunofluorescence of embryos.

Normal embryo developmental rates were assessed every 12 h as the percentage of embryos meeting the corresponding stage from the starting number of embryos seeded in Matrigel. E3.5 embryos were excluded if they did not recover blastocyst morphology within 24 h. (E3.5: Early Blastocyst, E4.0: Blastocyst, E4.5: Late Blastocyst, E5.0: Epiblast Rosette-like, E5.5: Egg Cylinder-like, E6.0: Pregastrula-like, E6.5: Early Gastruloid, E7.0: Mid Gastruloid, E7.5: Late Gastruloid [4, 16]). Bright-field channel wide-field images were imported into ImageJ 1.53a (Java 1.8.0_112 (64 bit)) to quantify embryo length. The longest embryonic body axis was measured as the embryo length using the line tool. All embryonic elongation rates were then plotted based on time points and conditions.

## Results

### The in vitro “sandwich” structure 3D model

**Fig. 1:**
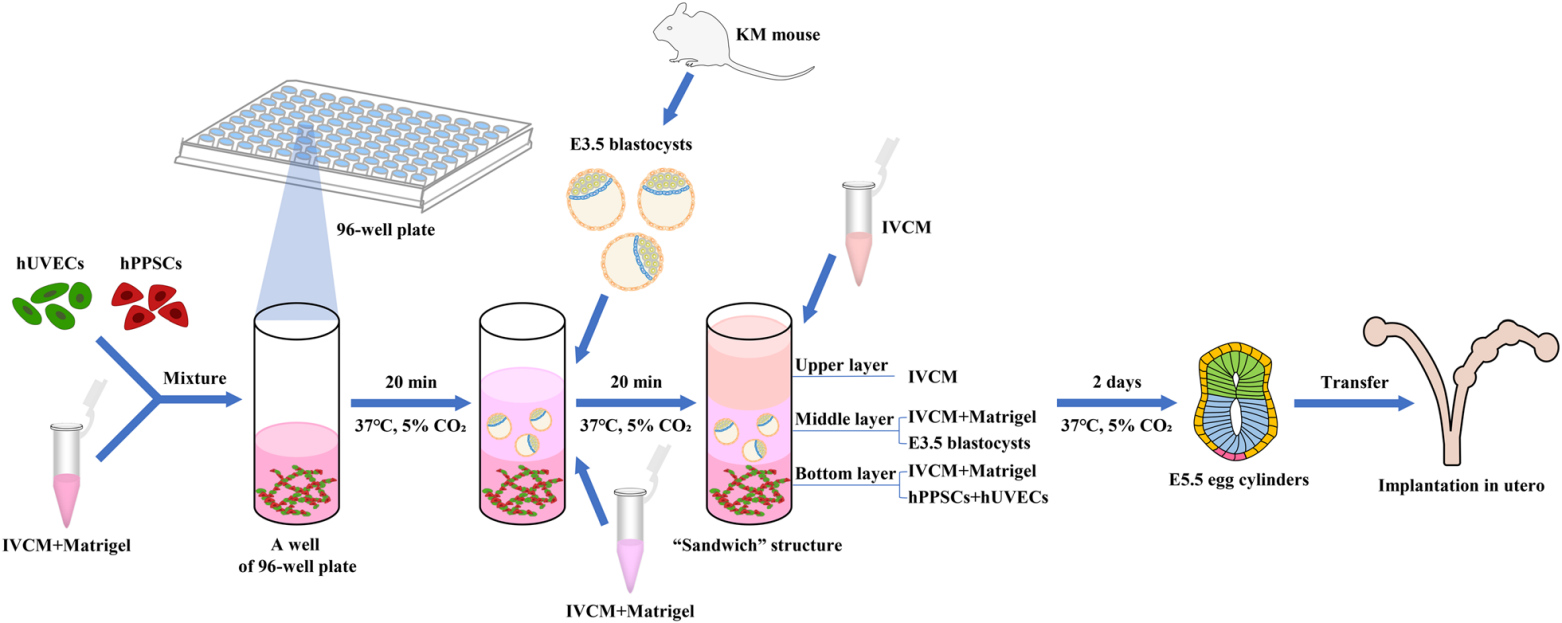
Schematic of the 3D “Sandwich” co-culture system with human placenta perivascular stem cells (hPPSCs) and human umbilical vein endothelial cells (hUVECs) supporting mouse embryo development from E3.5 to E7.5 in vitro

A protocol was prepared for in vitro embryo culture with a trilayer structure termed “sandwich.” The IVCM mixture of 23% high-glucose DMEM and 70% horse serum (and other components such as N-2 and B-27) was mixed with 30% Matrigel as described above to serve as the bottom and middle layers of the 3D culture environment. Additionally, supportive cells (including hPPSCs and hUVECs) were seeded into the bottom layer, E3.5 mouse blastocysts developed in the middle layer, and the upper layer of IVCM supplied nutrients to the embryos. Embryos cultured to E5.5 were re-implanted in utero after transplantation. The protocol model of cultured mouse embryos from E3.5 to E7.5 is recapitulated in Fig. 1.

### Characterization of hPPSCs and 3D co-culture with hUVECs on the Matrigel

**Fig. 2:**
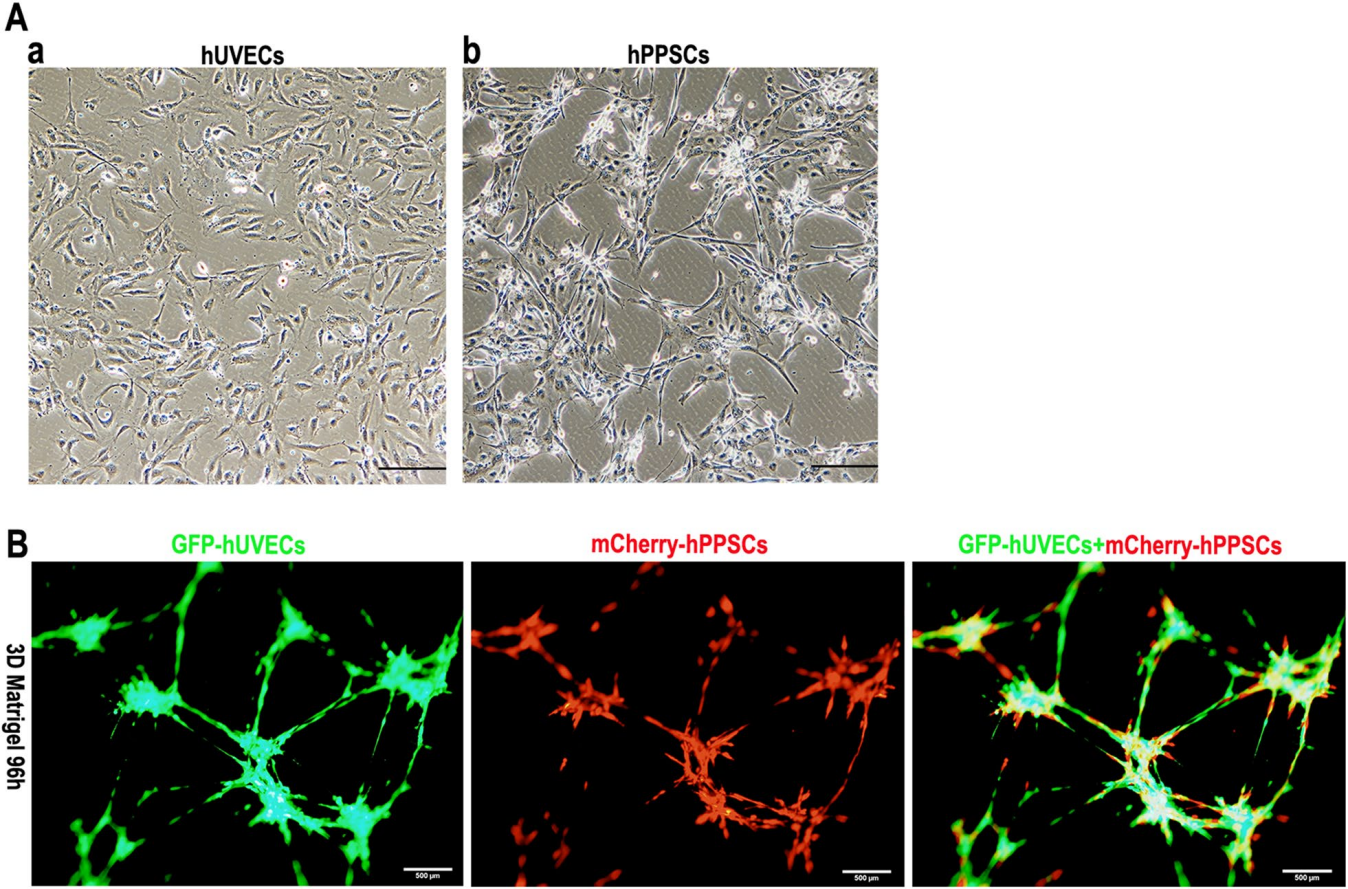
Human placenta perivascular stem cells (hPPSC) 3D co-culture with hUVECs on Matrigel. **A** The morphology of hUVECs and hPPSCs; scale bar, 100 µm. **B** GFP-hUVECs and mCherry-hPPSCs were co-cultured on Matrigel for 96 h; scale bar, 500 µm

hUVECs from human umbilical cord vein have a paving-stone shape (Fig. 2A, a); hPPSCs are triangle-like shape (Fig. 2A, b); mCherry-hPPSCs support GFP-hUVECs vascular stability on the 3D Matrigel (Fig. 2B); hPPSCs interact with hUVECs to form a vascular network in the 3D culture system with Matrigel.

### The IVCM + cells system supports embryonic development from E3.5 to E7.5 in the "sandwich" structure model in vitro

**Fig. 3:**
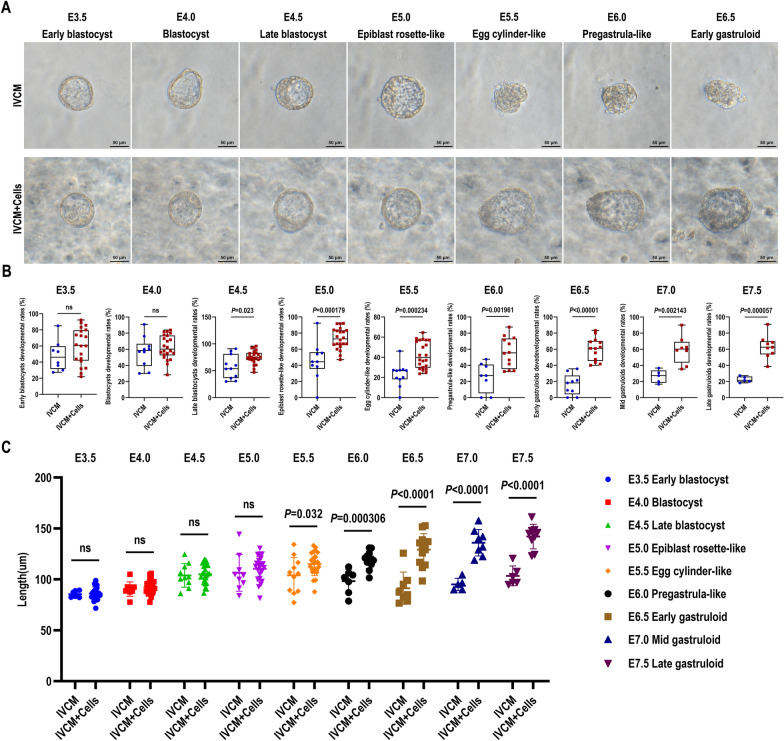
Development of mouse embryos in vitro from E3.5 to E7.5. **A** Bright-field images of mouse embryos cultured under the indicated conditions from E3.5 to E6.5. IVCM + Cells group refers to hPPSCs and hUVECs; scale bars, 50 µm. **B** Comparison of embryo development rates from E3.5 to E7.5 per 0.5 culture day. *n* = 5–23 for each group. *P* < 0.05 indicated statistical significance. **C** Comparison of embryo diameters from E3.5 to E7.5 per 0.5 culture day. *n* = 5–23 for each group. *P* < 0.05 indicated statistical significance

Two culture methods were designed to demonstrate that hPPSCs and hUVECs can support the development of mouse embryos: (1) the IVCM + Cells group containing hPPSCs and hUVECs in the bottom layer; (2) the IVCM group as a control. Embryos cultured in vitro were observed under an inverted microscope. Embryos in both groups had a typical blastoid morphology within the initial 36 h of in vitro culture (E3.5–E5.0), and the blastocoel cavities were also significantly enlarged. Remarkably, the size of blastocoel cavities was greater in the IVCM + Cells group, and the number of total embryonic cells dramatically swelled. Embryos in the IVCM + Cells group underwent lumenogenesis. They were continuously elongated after E5.0, formed egg cylinder-like structures at E5.5, and hatched from the zona pellucida. A pro-amniotic cavity was formed at E6.5, breaking the symmetry. These embryos were termed “early gastruloids.” However, embryos in the IVCM group exhibited abnormalities in subsequent development; they gradually atrophied, disintegrated, and died (Fig. 3A). Statistical analysis showed no significant differences in embryonic development rates between the two groups before E5.0. However, the IVCM + Cells group had 27.52% higher rates of epiblast rosette-like structures compared with the IVCM group (*n* (IVCM) = 10, *n* (IVCM + Cells) = 22, *P* = 0.000179) at E5.0; the rates of E5.5 egg cylinder-like structures were 18.81% higher (*n* (IVCM) = 11, *n* (IVCM + Cells) = 22, *P* = 0.000234); the rates of E6.0 pre-gastrula-like structures were 30.75% higher (*n* (IVCM) = 8, *n* (IVCM + Cells) = 12, *P* = 0.001961); the rates of E6.5 early gastruloids were 44.15% higher (*n* (IVCM) = 9, *n* (IVCM + Cells) = 13, *P* < 0.00001); the rates of E7.0 mid gastruloids were 33.31% higher (*n* (IVCM) = 5, *n* (IVCM + Cells) = 8, *P* = 0.002143); and the rates of E7.5 late gastruloids were 40.63% higher (*n* (IVCM) = 5, *n* (IVCM + Cells) = 9, *P* = 0.000057). All differences were statistically significant (Fig. 3B, Table 1, Additional file 1). In addition, the longest diameters of embryos at different developmental stages were measured to evaluate the size of embryos and the extent of elongation between the two groups. There were no significant differences in embryo diameters between the IVCM group and the IVCM + Cells group during the initial 36 h, whereas the diameters of the IVCM + Cells group were significantly higher than those of the IVCM group after E5.0. Embryo diameters of the IVCM + Cells group were 11.11 µm higher at E5.5 compared with the IVCM group (*n* (IVCM) = 11, *n* (IVCM + Cells) = 23, *P* = 0.032), 20.85 µm higher at E6.0 (*n* (IVCM) = 7, *n* (IVCM + Cells) = 12, *P* = 0.000306), 37.84 µm higher at E6.5 (*n* (IVCM) = 8, *n* (IVCM + Cells) = 13, *P* < 0.0001), 40.92 µm higher at E7.0 (*n* (IVCM) = 5, *n* (IVCM + Cells) = 8, *P* < 0.0001), and 38.58 µm higher at E7.5 (*n* (IVCM) = 6, *n* (IVCM + Cells) = 9, *P* < 0.0001). Each difference was statistically significant (Fig. 3C, Additional file 2).

**Table 1:** Percentages of normally developmental embryos cultured in IVCM and the IVCM + Cells system in vitro

Stage	IVCM	IVCM + Cells
E3.5 Early blastocyst (%)	48.31 ± 18.01	59.97 ± 21.19 (ns)
E4.0 Blastocyst (%)	56.74 ± 17.94	62.41 ± 14.73 (ns)
E4.5 Late blastocyst (%)	56.90 ± 20.92	75.11 ± 12.14 (ns)
E5.0 Epiblast rosette-like (%)	45.56 ± 22.44	73.07 ± 12.14 (*P* = 0.000179)
E5.5 Egg cylinder-like (%)	22.81 ± 11.04	41.62 ± 12.96 (*P* = 0.000234)
E6.0 Pregastrula-like (%)	25.50 ± 16.77	56.25 ± 18.23 (*P* = 0.001961)
E6.5 Early gastruloid (%)	16.23 ± 12.28	60.38 ± 13.62 (*P* < 0.00001)
E7.0 Mid gastruloid (%)	26.26 ± 7.20	59.57 ± 16.23 (*P* = 0.002143)
E7.5 Late gastruloid (%)	22.38 ± 3.32	63.01 ± 13.66 (*P* = 0.000057)

*n* = 5–23 for each group. All data were the mean ± SD. Statistical analysis was performed using one-way analysis of variance (ANOVA) with Tukey’s post hoc test. *P* < 0.05 indicated statistical significance. ns, Not significant. Cells in the IVCM + Cells group refer to hPPSCs and hUVECs

These results indicated that the 3D “sandwich” culture system promoted embryo development in vitro from E3.5 to E7.5. Mouse embryonic size, cell number, development rate, and elongation were significantly improved following hPPSC and hUVEC treatment.

### Expression of lineage markers in embryos cultured in vitro

**Fig. 4:**
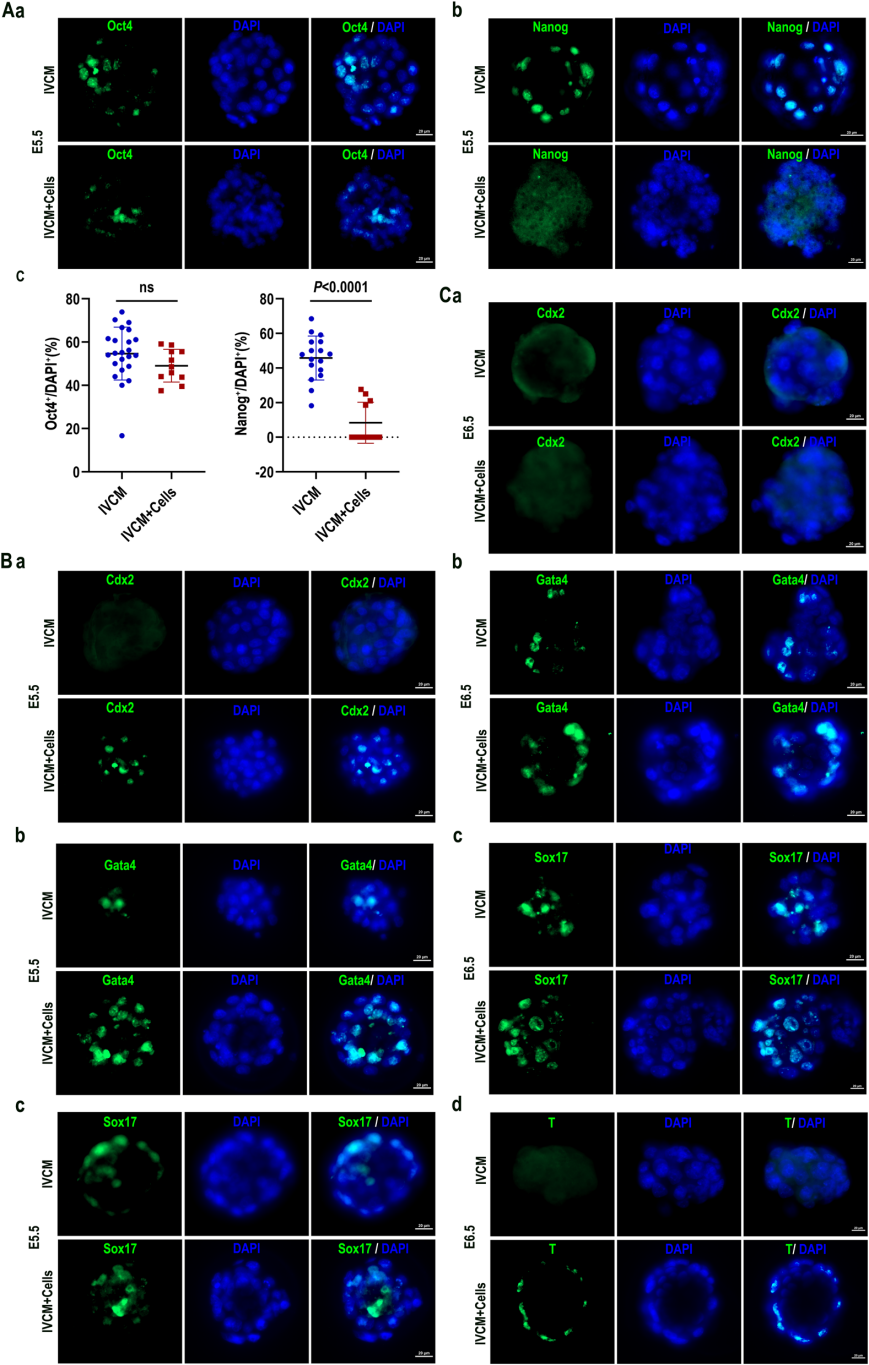
Expression of lineage markers in embryos cultured in vitro. **A** Immunofluorescence staining of Oct4 and Nanog from E5.5 embryos in the IVCM or IVCM + Cells group. Cells in the IVCM + Cells group are referred to as hPPSCs and hUVECs; scale bars, 200 µm. n = 11–23 for each group, *P* < 0.05 indicated statistical significance. **B** Immunofluorescence staining of Cdx2,Gata4 and Sox17 on E5.5 embryos in the IVCM or IVCM + Cells group; scale bars, 200 µm. **C** Immunofluorescence staining of Cdx2,Gata4,Sox17 and T on E6.5 embryos in the IVCM or IVCM + Cells group; scale bars, 200 µm

The expression levels of Oct4 and Nanog were examined on E5.5 embryos under different culture conditions by immunofluorescence to evaluate embryo developmental levels. Oct4 expressed both IVCM + Cells group (*n* = 11) and IVCM group (*n* = 23) (Fig. 4A,a). However, Nanog was only expressed in embryos from the IVCM group (*n* = 17), while there were few or no Nanog positive cells in embryos in IVCM + Cells group (*n* = 11) (Fig. 4A,b). We also quantified the numbers of OCT4^+^ and Nanog^+^ cells in embryos and analyzed the Oct4^+^/DAPI^+^ and Nanog^+^/DAPI^+^ ratios of embryos. Statistical analysis revealed no significant differences in the proportion of Oct4^+^ cells, (*n* = 23(IVCM), *n* = 11(IVCM + Cells), *P* > 0.01), the proportion of Nanog^+^ cells in embryos in the IVCM + Cells group was significantly higher than that in the IVCM group, (*n* = 17 (IVCM), *n* = 11 (IVCM + Cells), *P* < 0.0001) (Fig. 4A, c). The expression of Oct4 and Nanog provides clues to the developmental conditions of embryos since they are critical pluripotency factors during mouse embryo development: normally, Oct4 expression is persistent in implantation embryos, and Nanog expression is downregulated [47, 48]. The continued Nanog expression in E5.5 embryos suggested developmental anomalies in the IVCM group. Meanwhile, downregulated Nanog expression in the IVCM + Cells group suggested that these embryos were more similar to E5.5 natural embryos at the protein level. The extra-embryonic ectoderm (EXE), visceral endoderm (VE) and parietal endoderm (PE) have been identified in the stage of E5.5 [4, 49]. Gata4 is for VE markers, Sox17 is for PE markers, T/Bry is for mesoderm, and Cdx2 is for the maintenance multipotent state of the EXE [50]. At E5.5, the VE region is related to the distal visceral endoderm (DVE), and then the DVE migrates to the otic cylinder and is related to the anterior visceral endoderm (AVE) [51]. Based on the report, we examined the expression of the marker gene Cdx2 for EXE, Sox17 and Gata4 for VE and PE both in E5.5 and E6.5, as well as T/Bry for mesoderm in E6.5 embryos, which confirm the developmental capacity of the embryos in the 3D culture system. Cdx2 was only expressed in E5.5 embryos from the IVCM + Cells groups, but there was no expression in the IVCM groups (Fig. 4B, a). Gata4 and Sox17 were expressed in embryos from both the IVCM and the IVCM + Cells groups, but the number of positive cells in the IVCM + Cells groups was more than that of the IVCM groups (Fig. 4B, b–c). Different lineage markers of immunochemistry showed that E5.5 embryos in the IVCM + Cells groups have more developmental potency than that of IVCM groups, the embryos in IVCM groups were abnormal. It was surprising that there were no Cdx2 expression on E6.5 both IVCM and IVCM + Cells groups (Fig. 4C, a). The expression of Gata4 and Sox17 on E6.5 embryos from the IVCM + Cells groups was higher than that from IVCM groups (Fig. 4C, b–c). But T/Bry was only expressed in embryos from the IVCM + Cells groups, and there was no expression in IVCM groups (Fig. 4C, d). Since T is the marker of the primitive streak and nascent mesoderm, the above results suggest that the IVCM + cells system supports the embryo developing to E6.5 and undergoes gastrulation, but the function of IVCM is too limited. The results showed that 3D culture mouse embryos with Matrigel and cells have more linage capacity development.

### The developmental potential of transferred embryos in vivo

**Fig. 5:**
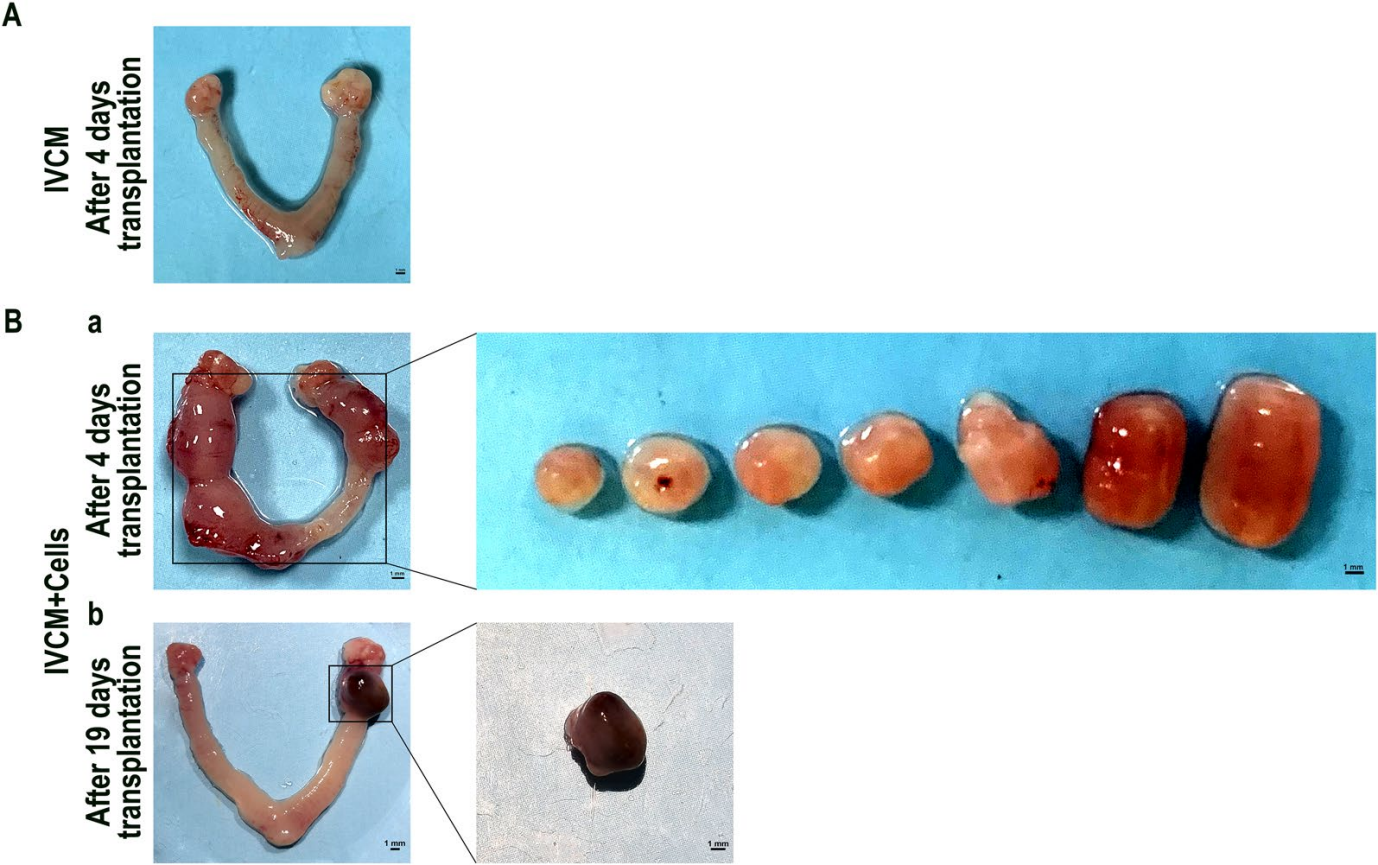
Potential development of transferred embryos in vivo. **A** Surrogate mouse uterus 4 days after embryo transfer with IVCM culture, Scale bars, 1 mm. **B** Surrogate mouse uterus 4 days and 19 days after embryo transfer with IVCM + Cells group. Cells in the IVCM + Cells group refer to hPPSCs and hUVECs. Scale bars, 1 mm

Embryos cultured for 5.5 days in vitro were transferred into E3.5 pseudopregnant mice, and the uterus was dissected later to explore the developmental potential of embryos in vivo under two culture protocols. None of the 15 transplanted embryos in the IVCM group were implanted *in utero* after transplantation 4 days (Fig. 5A), whereas 7 out of 15 embryos in the IVCM + Cells group were implanted at the uterine horn on the transplanted side, and the implantation rate was 46.67% after transplantation 4 days (Fig. 5B, a, Table 2). But, none of fetus have been survived after transplantation 14 days (Fig. 5B, b). These results suggested that E3.5 embryos in the IVCM group could develop to a certain stage, although they could not reimplant *in utero* after transplantation 4 days. This implied that they partially lost developmental potential. In contrast, the culture system with added hPPSCs and hUVECs simulated an early embryonic developmental environment in vivo*,* and the embryos cultured in this system could re-implant into the uterus and develop to a certain stage. This indicated that they exhibit better developmental potential in utero.

**Table 2:** Transfer results of embryos derived from the IVCM or IVCM + Cells group

Culture system	Number of embryos transferred	Number of implantation sites	Implantation sites/embryos transferred (%)
IVCM	15	0	0.00
IVCM + Cells	15	7	46.67

Cells in the IVCM + Cells group refer to hPPSCs and hUVECs

## Discussion

In vitro culturing of mouse embryos has been a research hotspot since the middle of the last century. Implantation is a crucial developmental event since the quality of the embryos determines whether subsequent development can be normally carried out until the neonatal mice are born. Embryoid models derived from embryonic stem cells can recapture a critical transformation during gastrulation [25, 26]. However, these models differ from natural embryos and rarely involve embryonic developmental events during implantation. This study devised a 3D “sandwich” culture system seeking to construct a vascularized microenvironment via the addition of supportive cells (hPPSCs and hUVECs) to the bottom layer of the Matrigel to promote the development of mouse embryos from the blastocyst (E3.5) to the gastrula (E7.5) stages. Activation of the Wnt signaling pathway in EBs contributes to the self-organization of ESC aggregates and the formation of the body axis [24]. Treatment with Act and CHI transforms round gastruloid aggregates into oval structures, and some pro-amniotic cavities are observed [13, 26]. Blastocysts are influenced by the vascularized microenvironment and start to polarize at E5.0, form the pro-amniotic cavity-like structure at E5.5, and trigger gastrulation, resembling the developmental processes of embryoids and natural embryos *in utero* [4]. A normal development rate is an essential indicator to estimate the quality of embryos and in vitro culture systems. An average of 30% of stem cell aggregates commence polarization and elongation to form gastruloids [26]. Our study showed no significant differences in the development rates between the protocols from E3.5 to E5.0. However, there were significant differences in the normal development rates within 48 h from E5.5 to E7.5. This accounted for the strong vitality of embryos that maintained normal development at the initial stage of in vitro culture. The development of embryos cultured in the IVCM system to the pre-gastrulation stage (E5.5) resulted in the embryonic cytoactivity gradually decreasing with a gradual decline in the growth of cell numbers in the embryo. This stagnation of development resulted in the majority of embryos in the IVCM group shrinking, dying, and ultimately disintegrating. However, embryos in the IVCM + Cells group showed higher gastrulation rates in the vascularized microenvironment compared with the IVCM group. We conjecture that there are two reasons for this discrepancy. First, previous studies were based on aggregates derived from mESCs, while the E3.5 embryos cultured in this study had higher development potential. Second, the vascularized microenvironment is formed by supportive cells that secreted factors required for embryonic development. It is widely acknowledged that embryo growth is completed by coordinated regulation of various factors. Vascular endothelial growth factor (VEGF) and fibroblast growth factor 2 (FGF2) are conducive to vascularization [52] and play vital roles in implantation. Simultaneously, leukemia inhibitory factor (LIF) and interleukin-11 (IL-11) are indispensable for implantation. Successful implantation is also affected by granulocyte–macrophage colony-stimulating factor (GM-CSF), interleukin-1 (IL-1), and interleukin-6 (IL-6) to some extent [53]. Fibroblast growth factor 2 regulates cell migration and neural differentiation during gastrulation [49, 54]. Factors secreted by supportive cells simulated certain conditions required for in vivo development; therefore, embryos cultured in the IVCM + Cells system still had higher development potential. Embryonic cell numbers increased, and embryos gradually underwent polarization and elongation to trigger the process of gastrulation. The polarization and elongation of embryos were quantified by measuring the longest diameters. The elongation of embryos in the IVCM + Cells group was higher than that in the IVCM group since the gastrulation stage was in accord with our observations. Nevertheless, the specific modes by which supportive cells impact embryos and the signaling pathways triggered by them require further exploration.

To date, various systems have been designed for in vitro embryo culture to simulate natural embryonic development in vivo. Some important developmental signals in embryos are absent when cultured under 2D conditions [55]. Rollers, circulators, and other devices were utilized to imitate the dynamic development environment [16, 22, 35]. This study established a 3D “sandwich” culture system by mixing a certain portion of Matrigel and IVCM to serve as the bottom and middle layers. Although a static system, the Matrigel ensured interactions between supportive cells and embryos. Additionally, we utilized the IVCM culture medium consisting of high-glucose DMEM, horse serum, N-2 supplement, and B-27 supplement based on a synthesis of the culture media used in previous studies [3, 11, 16, 44–46]. Horse serum can increase the number of embryonic cells and promote the formation and hatching of blastocysts, which facilitates embryogenesis [56]. N2B27 medium can induce and sustain naïve pluripotency in mouse embryos [57].

This study showed that an appropriate coagulation time of the Matrigel determined the quality of embryo development; prolonged or overly short coagulation may result in failure.

*Oct4* is a key gene in the pluripotency network during mouse embryonic development. It regulates cellular metabolic and biophysical properties to establish pluripotency in developing embryos [58] and affects the epiblast polarization and cavity formation of the epiblast rosette [4]. Thus, it participates in lumenogenesis and epithelialization [48]. Nanog is expressed in pluripotent stem cells and is downregulated during cell differentiation; it resembles Oct4 as a pluripotency protein [59]. Nanog expression arises in the late morula during the development of mouse embryos, and it is then confined to the ICM and late epiblast. However, Nanog expression is downregulated prior to implantation and further declines throughout the implantation stage. Oct4 expression is similar to Nanog expression, although it is significantly expressed in the epiblast of the egg cylinder at E5.5 [47, 48].

Oct4 was expressed in the IVCM and IVCM + Cells groups when embryos were cultured to E5.5 in vitro. This suggested that some embryonic cells were still pluripotent. In addition, Oct4 was significantly expressed in E5.5 natural embryos and other embryoids, such as ETS, ETX, and iETX embryos, and the expression position was similar to that in natural E5.5 embryos [11, 13, 28]. However, Nanog expression was significantly different between the two groups. The embryos in the IVCM + Cells group exhibited weak Nanog expression, which was the same as E5.5 embryos in vivo, and downregulation of Nanog expression before implantation is beneficial to restrict expansion of the epiblast and initiate egg cylinder formation [47]. It was demonstrated that E5.5 embryos cultured in the IVCM + Cells system had more similarity in terms of protein levels at the corresponding stage than those in the IVCM group. Furthermore, the normal expression of Nanog also facilitates further differentiation and development of embryos to prepare for embryo implantation into the uterus. In contrast, Nanog remained significantly and widely expressed in embryos from the IVCM group at E5.5. This restricts the differentiation of ectodermal cells into three germ layers [59], which may explain the developmental malformations observed in embryos after E5.0. The structural defects of E5.5 embryos in the IVCM group were also a failure factor in our transplantation experiments. In addition, high Nanog expression was not reported in other embryoids that are similar to E5.5 natural embryos.

The entire culture process, from the blastocyst stage to the birth of healthy mice in vitro*,* still needs to be completed owing to the complexity of the uterine environment. Therefore, we transferred embryos that were cultured in vitro for 48 h into the uterus of pseudopregnant mice to verify whether their developmental potential was affected. The transplantation of embryos cultured to the blastocyst stage in vitro successfully delivered mice [19], and blastoids can be implanted when transferred into pseudopregnant mice [37]. However, few studies transformed blastocysts at the implantation stage and monitored development after transfer. This study transferred embryos cultured from the blastocyst (E3.5) to pre-gastrula (E5.5) stages *ex utero* into pseudopregnant mice. Embryos cultured in the IVCM + Cells system still maintained the latent capacity to implant with the factors secreted by supportive cells. Embryos cultured in the IVCM system were deprived of implantation potential. There were no significant differences in the development rates from E3.5 to E5.5. Therefore, certain signaling pathways in the IVCM system could not be activated, resulting in a loss of development potential and implantation ability. Hence, it was proven that certain factors secreted by supportive cells are similar to the signals required for the implantation of natural embryos and contribute to the normal development of embryos in vitro.

We have developed an in vitro culture system for E3.5–E7.5 embryos. However, this system was incapable of *ex utero* development of embryos after E7.5, and the development rates of normal embryos also need to be improved. The other limitation of the study was the morphological differences observed between our embryoids and natural embryos. Given the changing maternal environment of developing embryos in the uterus, we still need to adjust the subsequent culture protocols to provide the corresponding developmental environment for embryos at different developmental stages. Notably, our control experiments demonstrated that hPPSCs and hUVECs played critical roles in the normal development of in vitro cultured embryos during implantation and post-implantation. However, further exploration is required to determine whether embryonic development is affected by the EVs of the supportive cells. Nevertheless, we established an in vitro co-culture system for mouse embryos from E3.5 to E7.5, and the cultured embryos expressed embryonic cell markers of the corresponding stage and could be implanted into the uterus after transplantation.

## Conclusion

The co-culture system can simulate the *in utero* environment of the implantation stage of mouse embryos to allow further study of the physical and molecular mechanisms involved in the critical process of embryo implantation and to facilitate further study of human embryos at the implantation stage.

### Supplementary Information

**Additional file 1**. Statistics of embryonic development.

**Additional file 2**. Statistics of embryonic diameter.

## Data Availability

The data and material are available from the lead corresponding author.

## Abbreviations

3D: Three-dimensional
Act: Activin A
ANOVA: Analysis of variance
AVE: Anterior visceral endoderm
BSA: Bovine serum albumin
Chi: CHIR99021
DAPI: 4′,6-Diamidino-2-phenylindole
DMEM: Dulbecco's modified Eagle’s medium
dpc: Days post-coitum
DVE: Distal visceral endoderm
E: Embryonic day
EBs: Embryoid bodies
endMSCs: Endometrial mesenchymal stromal cells
EPI: Epiblast cells
EPS: Extended pluripotent stem
ESCs: Embryonic stem cells
ETS embryos: ESC and TSC derived embryo
ETX embryos: ESC + TS + XEN embryos
EVs: Extracellular vesicles
EV-endMSCs: Extracellular vesicles derived from endMSCs
EXE: Extra-embryonic ectoderm
FGF: Fibroblast growth factor
FGF2: Fibroblast growth factor 2
GM-CSF: Granulocyte–macrophage colony-stimulating factor
hCG: Human chorionic Gonadotropin
hPPSCs: Human placenta perivascular stem cells
hUVECs: Human umbilical vein endothelial cells
ICM: Inner cell mass
iETX embryos: Induced ETX embryos
IL-1: Interleukin-1
IVCM: In vitro culture medium
iXEN cells: Inducible XEN cells
LIF: Leukemia inhibitory factor
MEM NEAA: Minimal essential medium non-essential amino acids
mESCs: Mouse embryonic stem cells
PE: Primitive endoderm
PGCs: Primordial germ cells
PMSG: Pregnant mare’s serum Gonadotropin
TE: Trophectoderm
TS: Trophoblast stem
VE: Visceral endoderm
VEGF: Vascular endothelial growth factor
XEN: Extra-embryonic endoderm
